# Effect of Individual and Selected Combined Treatments With Saline Solutions and Spent Engine Oil on the Processing Attributes and Functional Quality of Tomato (*Solanum lycopersicon* L.) Fruit: In Memory of Professor Leila Ben Jaballah Radhouane (1958–2021)

**DOI:** 10.3389/fnut.2022.844162

**Published:** 2022-04-28

**Authors:** Riadh Ilahy, Imen Tlili, Zoltán Pék, Anna Montefusco, Hussein Daood, Mohamed Azam, Mohammed Wasim Siddiqui, Thouraya R'him, Miriana Durante, Marcello Salvatore Lenucci, Lajos Helyes

**Affiliations:** ^1^Laboratory of Horticulture, National Agricultural Research Institute of Tunisia (INRAT), University of Carthage, Ariana, Tunisia; ^2^Horticultural Institute, Hungarian University of Agriculture and Life Sciences, Gödöllo, Hungary; ^3^Dipartimento di Scienze e Tecnologie Biologiche ed Ambientali (DiSTeBA), Università del Salento, Lecce, Italy; ^4^Institute of Horticultural Sciences, University of Agriculture, Faisalabad, Pakistan; ^5^Department of Food Science and Postharvest Technology, Bihar Agricultural University, Bhagalpur, India; ^6^Istituto di Scienze Delle Produzioni Alimentari (ISPA)-CNR, Lecce, Italy

**Keywords:** carotenoids, flavonoids, phenolic, radical scavenging activity, vitamin C, salt stress, soil pollution, tocopherols

## Abstract

The results showed that soil electrical conductivity, (EC2: 7 dS/m) increased soluble solids, lycopene content, total phenolic content, hydrophilic and lipophilic radical scavenging activities (HRSA and LRSA) by 14.2, 149, 20, 46.4, and 19.0%, respectively, compared with control. Under 0.5% spent engine oil (SEO), flavonoid content decreased by 21.7% compared with the control. HRSA and LRSA of fruits subjected to EC2/SEO1 treatment were, respectively, 45.9 and 35.5% lower than control. The a^*^/b^*^ ratio was positively and significantly (*P* < 0.01) correlated with β-carotene (R = 0.78), lycopene (R = 0.68), total vitamin C (R = 0.71), α-tocopherol (R = 0.83), γ-tocopherol (R = 0.66), HRSA (R = 0.93), LRSA (R = 0.80), and soluble solids (R = 0.84) suggesting that it may be a promising indicator of fruit quality in areas affected by such constraints. The research revealed that combined stresses induce responses markedly different from those of individual treatments, which strain the need to focus on how the interaction between stresses may affect the functional quality of tomato fruits.

## Introduction

Tomato (*Solanum lycopersicon* L.) is one of the most important vegetable crops in the world, ranking second just after potato. The fruits are excellent dietary sources of minerals, fibers, vitamins, and several antioxidants, principally the distinctive red pigment lycopene (1). This linear carotene is a powerful scavenger of free radicals, the major driving factor in the pathophysiology of various chronic and age-related diseases (2). Moreover, evidence is emerging on its role as a potential effector in the prevention and therapy of several disorders including spleen inflammation, neuropathy, and dyslipidemia, and also in exerting potential beneficial effects on skeletal muscle metabolism (3, 4). Besides lycopene, tomato also contains many other hydrophilic, and lipophilic antioxidants such as phenolic compounds (mainly phenolic acids and flavonoids), tocopherols, ascorbic, and dehydroascorbic acids contributing to protection against free radicals (5).

Salinization is a major abiotic factor limiting agricultural production worldwide. Salt pollution disturbs plant development by limiting its nutrient uptake and reducing the quality of the water available to the plant. It affects the metabolism of soil organisms and severely reduces soil fertility. High levels of salinity in soils provoke plant withering due to the increase in osmotic pressure and the toxic effects of salts (6).

The effect of saline water irrigation on tomato productivity and fruit quality has been well-defined, indicating that the thresholds of electric conductivity (EC) for the decrease of yield and plant growth are moderately high and differ among cultivars. Recently Ben Ali et al. (7), treating the cultivar (cv.) Rio Grande with salt solutions with EC of 3.5 and 7.0 deciSiemens per meter (dS/m), reported a significant reduction in plant height and root depth, but not in leaf area index, concluding that irrigation with saline is possible for tomato crop within adequate limits. Indeed, within defined EC values, an improvement of ripe tomato fruit quality and taste has been reported under salinity (8, 9). In addition to soil salinization, several pollutants increasingly threaten agricultural areas affecting crop growth, production, and quality (10). More than 50% of the main soil contaminants are petroleum hydrocarbons deriving primarily from accidental spillage during extraction, transportation, and distribution of petroleum and petroleum derivatives, but also from the illicit tapping from pipelines or dumping of spent products. These criminal activities represent a global threat concerning both developing and industrialized countries (11, 12). Actually, between 2010 and 2016, more than 20 pipeline liquid spillage incidents have been recorded each year in Europe and 399 in the United States, while 465 intentional unlawful discharge acts were reported in Italy between 2011 and 2019, which generally resulted in soil pollution (13). In the ScienceDirect database (www.sciencedirect.com), using oil spill and agriculture as keywords, the number of published research items has increased sharply from 175 to 1,353 between 1998 and 2021, suggesting the importance of carefully focusing on the deleterious effect of such problems. In particular, soil and surface water contamination with spent engine oil (SEO) is becoming a prevalent environmental issue in most developing countries (14). SEO reduces soil aeration causing root stress and chlorosis of leaves and leads to an impairment of soil microbial community with deleterious effects on fertility and crop production potential. These deleterious effects have been thoroughly documented on various crops, including maize, peanut, cowpea, bean, gombo, green amaranth, sponge gourd, fluted pumpkin, and pepper (14–20).

To the best of our knowledge, there is no detailed data regarding the effect of salinity and SEO on tomato fruits quality. Thus, this research was carried out to fill this knowledge gap by assessing the impact of individual and combined treatments with saline water and SEO on the main processing traits and functional quality of ripe tomato fruits of the cv. Rio Grande.

## Materials and Methods

### Chemicals

2,20-Azinobis (3-ethylbenzothiazoline-6-sulphonic acid) diammonium salt (ABTS), ascorbic acid, Rutin, Gallic acid, butylated hydroxytoluene (BHT), NaOH, NaNO_2_, AlCl_3_ were obtained from Sigma–Aldrich, Chemical Co., Milan. Other reagents were of analytical grade and purchased from Carlo Erba (Milan, Italy).

### Plant Material and Treatments

**Figure 1:**
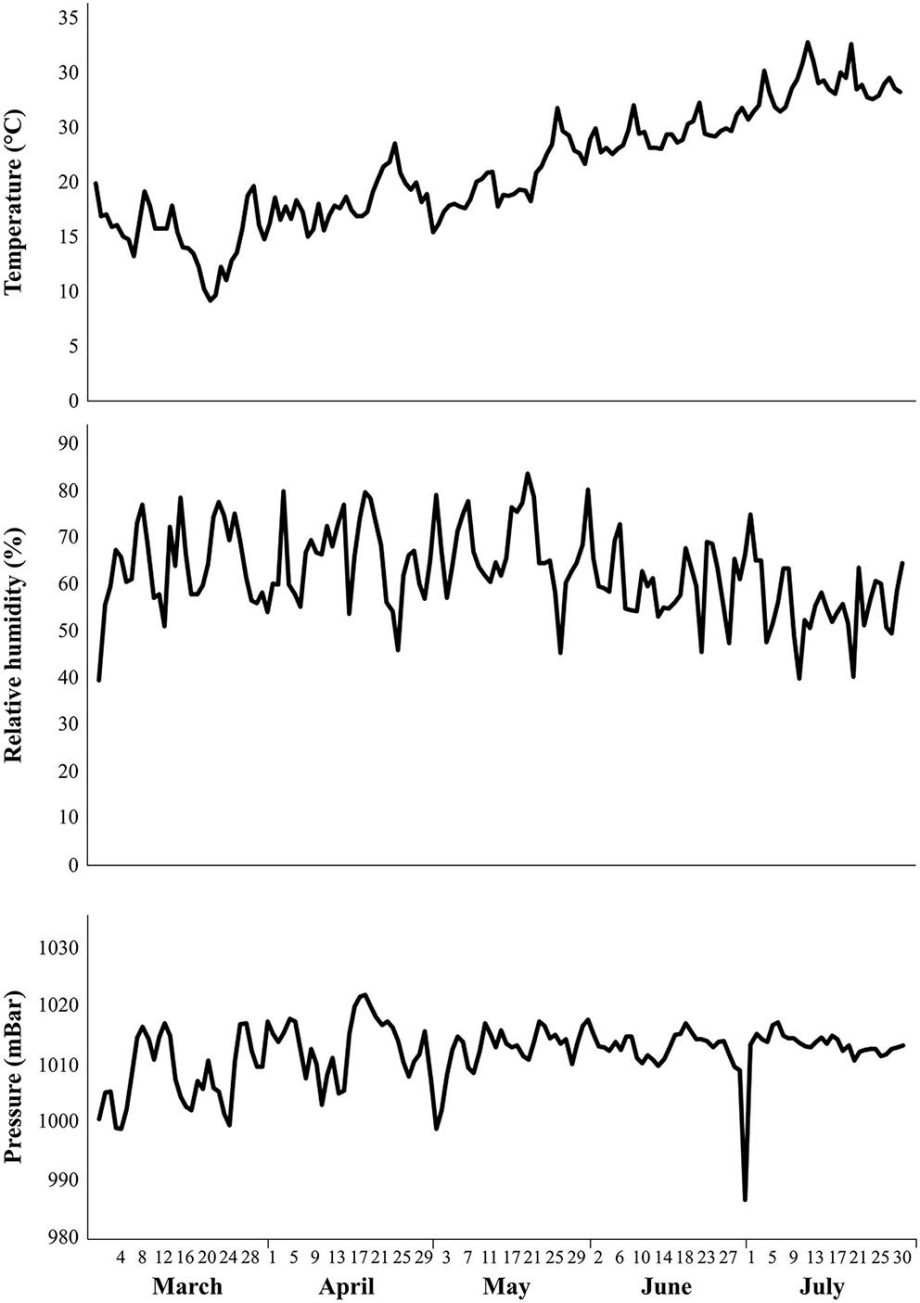
Temperature (°C), relative humidity (%), and atmospheric pressure (mBar) data were recorded by the weather station of the National Agricultural of Tunisia (Lat 40°20′ N, Lon 18°07′) of the University of Carthage. The reported values refer to 2018 and cover the entire tomato plant growing season (March–July).

Certified tomato seeds of the cv. Rio Grande was provided by the germplasm bank of the National Agricultural Research Institute of Tunisia. Rio Grande is a determinate open-pollinated highly productive (97.0–145.4 t/ha) tomato cv. widely grown in Tunisia. It produces large fruits suitable for both fresh consumption and processing with an average weight ranging from 77.0 to 81.2 g and soluble solids content between 5.2 and 5.9°Brix (5). Sowing was carried out on plastic trays in February 2018. A total of four-week-old seedlings were individually transplanted into 20 L pots containing a clay-loamy substrate with 246.2 g/kg clay, 160 g/kg loam, and 290.7 g/kg sand and high levels of the calcareous substance, with pH (7.72), EC (0.19 mS/cm), mineral and organic composition suitable for tomato cultivation. The pots were placed under natural meteorological conditions (Figure 1) in the experimental field of the University of Carthage, with additional watering as required by the experimental design.

A total of 10 days after transplant, seedlings were exposed to seven different treatments (30 plants/treatment): two salinity levels (EC1: 3.5 dS/m and EC2: 7.0 dS/m, equivalent to 5.46 and 10.93 g NaCl/L, respectively); two levels of SEO [SEO1: 5 ml SEO/L (0.5%) and SEO2: 10 ml SEO/L (1%)], two combinations of saline and SEO levels (EC1/SEO2: 3.5 dS/m, 1% SEO and EC2/SEO1: 7dS/m, 0.5% SEO) and a common control treatment (0.4 dS/m EC and no SEO application). SEO was applied directly around the root zone of tomato plants before irrigation 2–3 times a week throughout seven weeks. Some combined treatments were not considered in the present research as preliminary tests showed that EC2/SEO2 treatment led to plants severely affected in growth parameters and leaves' cover with no fruit set, while EC1/SEO1 treatment gave results similar to SEO1, at least in terms of yield, average fruit weight and visual appearance of fruit cross-sections. The irrigation management was based on monitoring the soil moisture content using a BIOS probe measuring simultaneously the moisture content and temperature. Irrigation was performed manually until the water content of the used substrate reached the field capacity value (34%).

### Fruit Sampling

Tomato fruits were collected from each plant at the red stage of ripeness and yield per plant (yield/plant) was determined. A total of 18–20 healthy fresh ripe-red tomato fruits were collected from each bloc and quickly carried to the laboratory. The sampling was performed in triplicate when the red-ripe stage was attained. Bright red-ripe fruits were selected, externally washed with deionized water, cut into small pieces, and homogenized in a blender (Waring Laboratory Science, Torrington, CT, US). The homogenates were frozen at −20°C and used to determine the content of carotenoids, total phenols, flavonoids, vitamin C as well as HRSA and LRSA within the following days to prevent nutrient degradation.

### Determination of Total Soluble Solids, Titratable Acidity, and Color Indexes

Brix of freshly prepared juice was used to measure the soluble solid content of tomato fruits. For this reason, some drops of filtered juice were placed on the prism of an Atago PR-100 digital refractometer equipped with automated temperature correction. Titratable acidity was calculated as a percentage of citric acid following titration of the diluted juice using 0.1 M sodium hydroxide solution until reaching 8.1 pH. A Minolta CR-400 (Minolta Corp., Osaka, Japan) was used to estimate the redness a^*^ and b^*^ yellowness color indexes and the a^*^/b^*^ ratio was consequently calculated.

### Analysis of Carotenoid Content

Carotenoids were determined according to the protocol of Daood et al. (21) slightly modified. Briefly, tomato homogenate (2.5 g aliquots) was crushed in a crucible mortar with quartz sand. Subsequently, 20 ml of methanol was added for 1–2 min, and the upper layer was poured into an Erlenmeyer flask. A volume of 50 ml of dichloroethane was then mixed with 10 ml of methanol in a graduated cylinder and mixed softly before and after adding some distilled water drops. The mixture was filtrated in a separating funnel and allowed to evaporate at 70°C until complete evaporation. A volume of 5 ml of analytical grade methanol was mixed with 5 ml of pigment eluents and poured into the same flask then mixed gently, sonicated, filtered using a 0.22 μm membrane syringe, and finally injected into the HPLC (Hitachi Chromaster, Tokyo, Japan) system consisting of a 5,110 Pump, a 5,210 Auto Sampler, a 5,430 Diode Array detector, and a 5,440 FL detector.

### Determination of Total Phenolic and Flavonoid Contents

The procedure of Martínez-Valverde et al. (22) was used for the extraction and quantification of total phenolics content. In total, 5 ml of methanol 80% and 50 μl of 37 % HCl were mixed with 0.3 g of tomato homogenate and extracted for 2 h at 4°C under 300 rpm then centrifuged for 20 min at 10,000 g. The Folin–Ciocalteu reagent was used with a 50 μl supernatant sample and the absorbance was measured at 750 nm using a Cecil BioQuest CE 2501 spectrophotometer (Cecil Instruments Ltd., Cambridge, UK), and total phenolics content was expressed as mg of gallic acid equivalent (GAE)/kg fw. Moreover, the procedure of Asami et al. (23) was used to correct the obtained values due to the interference of sugars with phenolic compounds.

Zhishen et al. (24) described a method that was used for the determination of flavonoids content. For this reason, a sample of 0.3 g was extracted with methanol and a volume of 50 μl was diluted with distilled water to attain 0.5 ml, and 30 μl of 5% NaNO_2_ was poured and 60 μl of AlCl_3_ (10%) and 200 μl of NaOH (1 M) were added after 5 and 6 min, respectively. The wavelength 510 nm was used to measure the absorbances of the samples using a Cecil BioQuest CE 2501 spectrophotometer (Cecil Instruments Ltd., Cambridge, UK) and the results were expressed as mg of rutin equivalent (RE)/kg fw.

### Determination of Total Vitamin C Content

The extraction and quantification of total vitamin C (AsA + DHA) content were carried out according to Kampfenkel et al. (25) on triplicate samples (0.2 g) of homogeneous tomato juice. The absorbance was read at 525 nm in a spectrophotometer (Cecil BioQuest CE 2501) and expressed as mg/kg fw. The linear reading of the standard curve was from 0 to 700 μmol AsA.

### Determination of Tocopherol Content

The procedure of Abushita et al. (26) was adopted for the extraction of tocopherols using n-hexane. The separation was performed on Nucleosil 5 mm (250 4.6 mm i.d.) with a mobile phase consisting of 99.5:0.5 n-hexane: ethanol and detected at 295 and 320 nm as the excitation and emission wavelength, respectively, as outlined in Duah et al. (27). α, β, γ, and δ isomers (from Sigma-Aldrich Ltd., Budapest, Hungary) were used for the identification of tocopherols.

### Determination of the Radical Scavenging Activity

The radical scavenging activity of the hydrophilic and lipophilic fractions (HRSA and LRSA, respectively) was evaluated utilizing the TEAC assay. The method outlined by Miller and Rice-Evans (28) is widely used for the determination of the radical scavenging activity of fruit, vegetables, and processed products due to its reproducibility and high fidelity across complex matrices. The extraction of the antioxidants from the hydrophilic and lipophilic fractions was performed on 0.3 g of the tomato sample using methanol (50%) acetone (50%), respectively, at 4°C under constant shaking (300 rpm) during 12 h. Tomato homogenate samples were centrifuged for 7 min at 10,000 g, supernatants were used for the measurement of the radical scavenging activity at 734 nm in a Cecil BioQuest CE 2501 spectrophotometer (Cecil Instruments Ltd., Cambridge, UK). Radical scavenging activity was calculated and expressed as μM of Trolox/100 g of fw.

### Statistical Analysis

The variations affecting the processing and functional quality of tomato fruits under the applied treatments were evaluated by one-way ANOVA. When a significant difference was detected, the means were compared using the Duncan test (*P* <0.05). All the statistical comparisons were performed using version 6.1 of SAS software (IBM SPSS Statistics for Windows, Version 20.0. Armonk, NY: IBM Corp., NC, USA). Correlations were examined using Pearson's correlation coefficient (r).

## Results and Discussion

### Processing Attributes

The main processing attributes (yield/plant, average fruit weight, total soluble solids, titratable acidity, and a^*^/b^*^ ratio) of the red-ripe fruits harvested from tomato plants grown under control conditions or exposed to the individual and combined treatments with saline water and SEO are reported in Table 1 and expressed as percent variation with respect to the control in Table 2.

**Table 1:** Main processing attributes (yield/plant, average fruit weight, titratable acidity, total soluble solids and a^*^/b^*^ ratio) of red-ripe tomato fruits (cv. Rio Grande) harvested from plants subjected to control conditions (0.4 dS/m EC, no SEO), saline stress (EC1, 3.5 dS/m; EC2, 7 dS/m), spent engine oil stress (SEO1, 0.5%; SEO2, 1%), and saline/SEO combinations (EC1/SEO2, 3.5 dS/m/1% SEO; (EC2/SEO1, 7 dS/m/0.5% SEO).

**Traits/treatments**	**Yield/plant (g)**	**Average fruit weight (g)**	**Titratable acidity (%)**	**Soluble solids (**°**Brix)**	a*/b*
Control	1,201.7 ± 52.5 a	90.0 ± 3.5 a	0.37 ± 0.004 d	5.6 ± 0.06 c	1.14 ± 0.03 c
EC1	875.7 ± 12.9 b	82.33 ± 3.4 a	0.43 ± 0.05 d	6.2 ± 0.09 ab	1.41 ± 0.04 b
EC2	808.3 ± 18.8 b	73.33 ± 2.02 b	0.57 ± 0.006 c	6.4 ± 0.115 a	1.62 ± 0.07 a
SEO1	716.7 ± 8.9 c	64.33 ± 2.1 c	0.56 ± 0.01 c	6.03 ± 0.03 b	1.23 ± 0.1 c
SEO2	631.4 ± 13.3 d	59.66 ± 3.3 cd	0.63 ± 0.002 bc	5.43 ± 0.04 c	1.21 ± 0.05 c
EC1/SEO2	570.1 ± 5.8 d	53.33 ± 1.8 de	0.70 ± 0.03 b	5.16 ± 0.02 d	0.93 ± 0.02 d
EC2/SEO1	466.0 ± 11.5 e	48.66 ± 0.88 e	0.86 ± 0.03 a	5.36 ± 0.1 cd	0.85 ± 0.04 d

*Values are expressed as the mean ± SD of three independent experimental replicas (n = 3)*.

*Different letters in the same column indicate significant differences between means according to the Duncan's test at the 5% level*.

**Table 2:** Percent variation compared to the control in yield/pl. (Y/pl.), average fruit weight (Avg. fruit wt.), total soluble solids (TSS), titratable acidity (TA), a^*^/b^*^ ratio, β-carotene (β-Crt), lycopene (Lyc), total phenolic compounds (TPC), total flavonoids (TF), total vitamin C (TVC), α-, β-, and γ-Tocopherol (α-, β- and γ-T), hydrophilic radical scavenging activity (HRSA) and lipophilic radical scavenging activity (LRSA) under the different applied treatments and their combinations.

**Traits/ treatments**	**Y/pl**.	**Avg.fruit wt**.	**TSS**	**TA**	a*/b*	**β-Crt**	**Lyc**	**TPC**	**TF**	**TVC**	**α-T**	**β-T**	**γ-T**	**HRSA**	**LRSA**
**EC1**	−27.1%	−8%	10%	15%	23%	31%	98%	−17%	−5%	47%	31%	93%	18%	16%	19%
**EC2**	−32.8%	−18%	14%	53%	40%	49%	149%	20%	−11%	66%	26%	nd	26%	26%	19.3
**SEO1**	−40.3%	−28%	7%	51%	7%	47%	123%	−7%	−21%	95%	−56%	nd	−24%	−28%	−7%
**SEO2**	−47.5%	−33%	−29%	69%	6%	90%	37%	−8%	−0.3%	−14%	−19%	nd	−86%	−16%	7.1%
**EC1/SEO2**	−52.6%	−40%	−77%	89%	−18%	17%	43%	6%	−2%	−27%	−2%	nd	nd	−17%	−21%
**EC2/SEO1**	−60.9%	−46%	−41%	131%	−24%	−30%	29%	21%	−2%	−31%	3%	nd	nd	−26%	−35%

*Green cells identify increased changes, yellow cells identify reductions*.

*nd, not detected*.

All the evaluated processing attributes were significantly affected by treatments (*P* <0.05). Salinity and SEO applied individually and in combination, resulted in 27.1–32.7%, 40.3–47.5%, and 52.6–60.9% reduction in yield/plant, respectively, which decreased from the mean yield of 1,201.7 g/plant in the non-treated plant, down to only 466.0 g/plant upon combined exposure to 7 ds/m EC and 0.5% SEO (EC2/SEO1). Similarly, we noticed a reduction of 8–18%, 28–33%, and 40–46% in the average fruit weight, respectively, which decreased from the mean value of 90 g of a control sample; down to 48.66 g under 7 ds/m EC and 0.5% SEO combined exposure (EC2/SEO1). This may result from a drop in water balance/uptake determined by the reduction of soil water potential due to the increase of soluble salts and/or the decrease of soil water holding capacity promoted by SEO (29, 30). The treatments distinctively affected total soluble solids of tomato fruits: EC1, EC2, and SEO1, applied individually, resulted in a 7–14% increase, while, individual application of 1% SEO (SEO2) and both combined treatments (EC1/SEO2; EC2/SEO1) caused a significant (29–77%) decrease compared with the control. According to Botella et al. (8), salinity significantly increases total soluble solids and titratable acidity stimulating the synthesis of soluble sugars (glucose and fructose) and organic acids. Consistently, Yurtseven et al. (31) reported an increase in total soluble solids attaining 7.51 and 10.4 °Brix under 5 and 10 dS/m, respectively, compared with 5.43 °Brix of control fruits of the tomato cv. H2274-Oturak. Sugar and sugar derivative synthesis/accumulation may contribute to satisfy the increasing energy demand required to tolerate the adverse stress conditions and/or, acting as osmolytes, to protect cells and sustain a minimal osmotic balance under water stress conditions determined not only by salinity but also by pollution with high molecular weight polycyclic aromatic hydrocarbons (PAH) (29, 30). In this regard, Molina and Segura (32) reported that sugar metabolisms are activated by the presence of PAH with a consequential increase of glucose, mannose, galactose, raffinose, galactinol, melibiose, and sucrose contents in plant tissues. A decrease in fruit quality and sugar content was, instead, reported in peppers grown in hydroponic systems under a glasshouse exposed to 5 mS/cm salinity possibly determined by the enhancement of respiration rate of stressed fruits (33, 34), confirming that the responses to salinity stress are species specific, or even variety dependent.

With regard to SEO, no literature data are available on its effects on the total soluble solids of tomatoes, neither if applied individually, nor in combination with other stresses. Nevertheless, the observed negative effect on total soluble solids of 1% SEO treatment (SEO2), further amplified by the coexistence of saline stress, can probably be explained by a direct or indirect toxicity effect of the pollutant on the central carbon metabolism of plants. Indeed, SEO contains heavy metals [including lead (Pb), cadmium (Cd), arsenic (As), zinc (Zn), and copper (Co)] which interfere with physiological, biochemical, and molecular processes of living systems (35, 36).

EC and SEO treatments, individually and combined, resulted, respectively, in a 15–53%, 51–69%, and 89–131% increase of titratable acidity compared to control tomato fruits (Table 2), with values ranging from 0.37% (control), up to 0.86% (EC2/SEO1). The observed increase is possibly related to an active accumulation of ions and organic acids produced by the plant under stress conditions. Alterations in the relative concentrations of tricarboxylic acid cycle intermediates, resulting in increased citrate and malate levels but α-ketoglutarate, fumarate, oxaloacetate, pyruvate, and succinate decrease, were reported in response to PAH as a consequence of the overexpression or downregulation of the enzymes of the Krebs cycle and of the mitochondrial respiratory chain (32).

**Figure 2:**
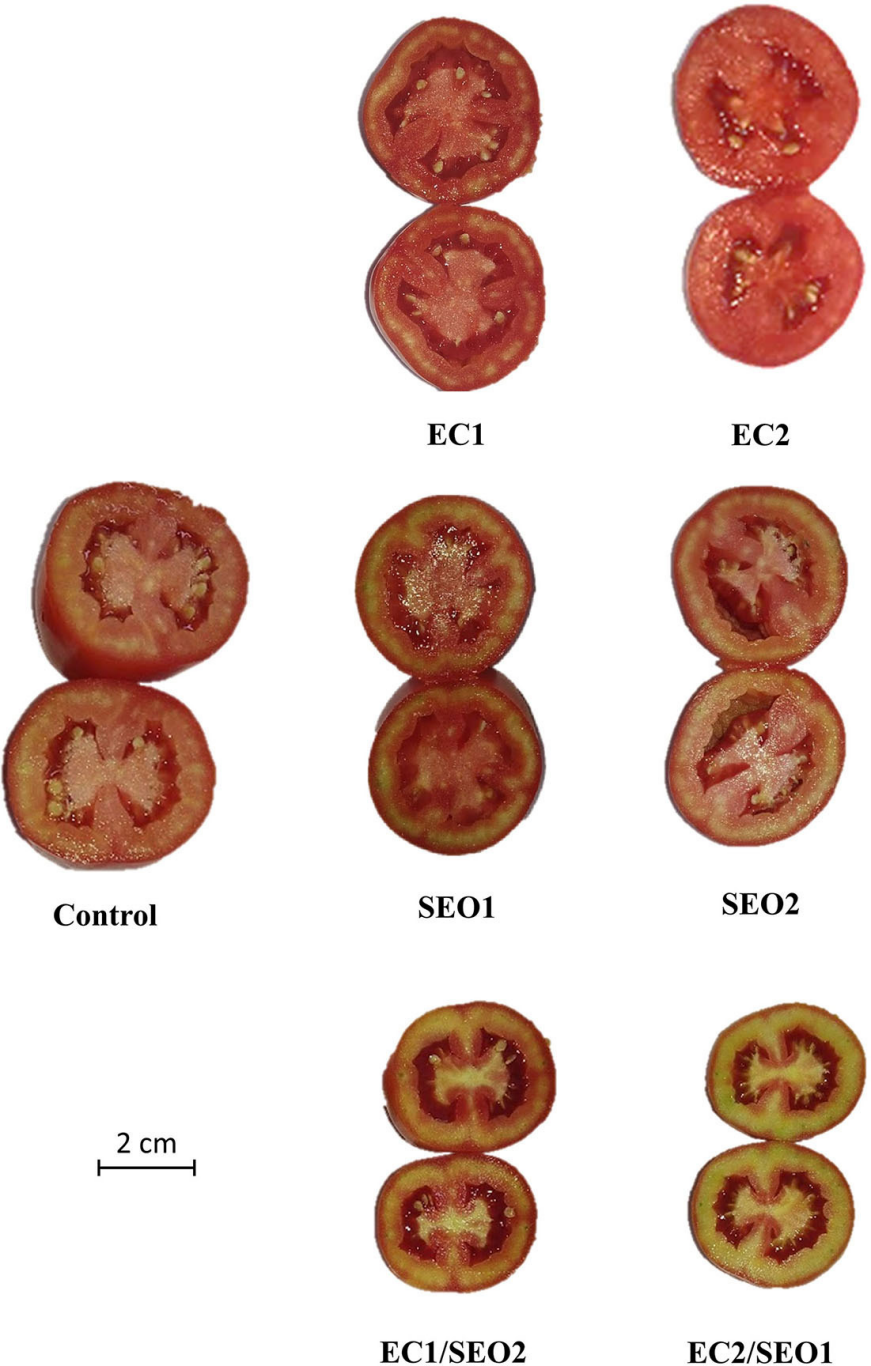
Transverse sections of tomato fruits under control (C, 0.4 dS/m), saline (EC1, 3.5 dS/m; EC2, 7 dS/m), spent engine oil stress (SEO1, 0.5%; SEO2, 1%) and the combinations (EC1/SEO2, 3.5 dS/m EC/1% SEO; EC2/SEO1, 7 dS/m EC/0.5% SEO).

The a^*^/b^*^ ratio is a suitable parameter to characterize the quality and maturity of tomato fruits (5, 37). EC and SEO treatments applied separately determined, respectively, a 23–40% and 6–7% increase in the a^*^/b^*^ ratio compared with the control, while the combined treatments prompted an 18–24% decrease (Table 2). Accordingly, an evident discoloration of the mesocarp characterizes the cross-sections of tomato fruit samples grown under EC1/SEO2 and EC2/SEO1 (Figure 2).

Stress interactions can be classified as (1) additive, when the response is proportional to the sum of the single applied stresses; (2) synergistic, when it overcomes the sum of the individual stresses; (3) idiosyncratic, when the outcome differs significantly from the stresses applied individually and (4) dominant when the response is similar to that induced by one of the stresses applied individually (38). Our findings hint at differences in the responses to the two assayed stress combinations for the same trait. For average fruit weight and total soluble solids, the response to EC1/SEO2 (3.5 dS/m EC, 1% SEO) and EC2/SEO1 (7 dS/m EC, 0.5% SEO) was, respectively, dominated by SEO and idiosyncratic. However, both stress combinations gave additive responses for titratable acidity, but idiosyncratic for a^*^/b^*^ ratio.

### Carotenoid Content

**Figure 3:**
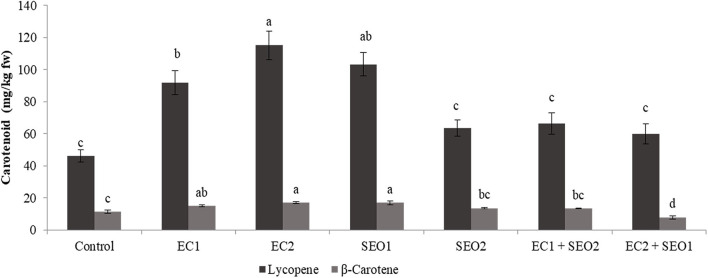
Lycopene and β-carotene content (mg/kg fw) of tomato fruits under control (C, 0.4 dS/m), saline (EC1, 3.5 dS/m; EC2, 7 dS/m), spent engine oil stress (SEO1, 0.5%; SEO2, 1%) and the combinations (EC1/SEO2, 3.5 dS/m EC/1% SEO; EC2/SEO1, 7 dS/m EC/0.5% SEO). Values are means (*n* = 3).

Ordinary red tomato fruit contains up to 200 mg of lycopene per kg of fresh weight (fw), together with much lower contents of β-carotene and other carotenoids, though with large genotype-associated variations (1, 5). In the pulp and peels of ripe Rio Grande tomatoes grown in an open field, the concentration of lycopene was approx. In total, 100.9 and 423.7 mg/kg fw, respectively (39), while the β-carotene level in the entire fruit homogenate was below 7 mg/kg fw (5). In this study, the level of lycopene in the control fruits was halved with respect to our previous report, while β-carotene remained almost unchanged (46.2 and 8.0 mg/kg fw, respectively) possibly due to the different growing conditions. EC and SEO treatments, either applied individually or combined, prompted significant (*P* <0.05) variations in the contents of both lycopene and β-carotene, which reached concentrations up to 115.1 and 17.0 mg/kg fw, respectively (Figure 3). In particular, compared with the control, the lycopene concentration was increased by 48–149% and 37–123% by individual EC and SEO treatments, respectively, and 29–43% by the stress combinations. Similarly, β-carotene levels were increased by 31–49% and 47–90% by the individual EC and SEO treatments, respectively. However, a 17% increase was observed under EC1/SEO2 conditions, while EC2/SEO1 treatment determined a 30% decrease in β-carotene, suggesting idiosyncratic responses. In agreement, a general improvement of tomato fruit quality attributes (lycopene, phenols, ascorbic acid, hydrophilic, and lipophilic antioxidant activities) was reported by Sellitto et al. (40) under 6.0 mS/cm soil EC, as well as in pepper (cv Friariello) fruits grown under 4.4 mS/cm EC in a nutrient film technique hydroponic system. Moles et al. (41) and Borghesi et al. (42) reported that tomato carotenoids exhibited a genotype-dependent trend in response to salinity. However, Serio et al. (43) found that salinity did not affect the lycopene content. Similarly, in the fruits of the tomato cv Boludo F1 grown under hydroponic conditions, phytoene, phytofluene, lycopene, and lutein contents were unaffected by salinity (7.8 dS/m), unlike β-carotene which was increased (8). Sumalan et al. (9) found that tomatoes grown under soil ECs over 6.5 dS/m exhibited moderate-to-high concentrations of various antioxidants including lycopene, phenolics, and ascorbic acid, and also higher total antioxidant activity, highlighting the important role of secondary metabolites in the process of stress adaptation. The discrepancy among the reported studies might be related to genotypic differences in the resistance/tolerance to different stresses, soil type, and several other environmental and agronomic factors. A decline in the content of chlorophyll and leaf carotenoids was observed following irrigation of tomatoes with industrial wastewater highly contaminated with heavy metals (44) and also in the presence of toxic levels of Cd, probably due to inhibition of the carotenoid biosynthetic pathway and severe oxidative stress induction (45–47). It has been also reported that cadmium inhibited the photosynthetic activity of photosystem II by 60% but was ineffective on photosystem I. Muzolf-Panek et al. (48) reported that the levels of lycopene and vitamin C decreased in tomato cvs Emoticon F1 and Alboney F1 fruits grown in hydroponics under increasing Mn concentrations (0–19.2 mg/dm^3^).

### Total Phenolic and Flavonoid Contents

**Figure 4:**
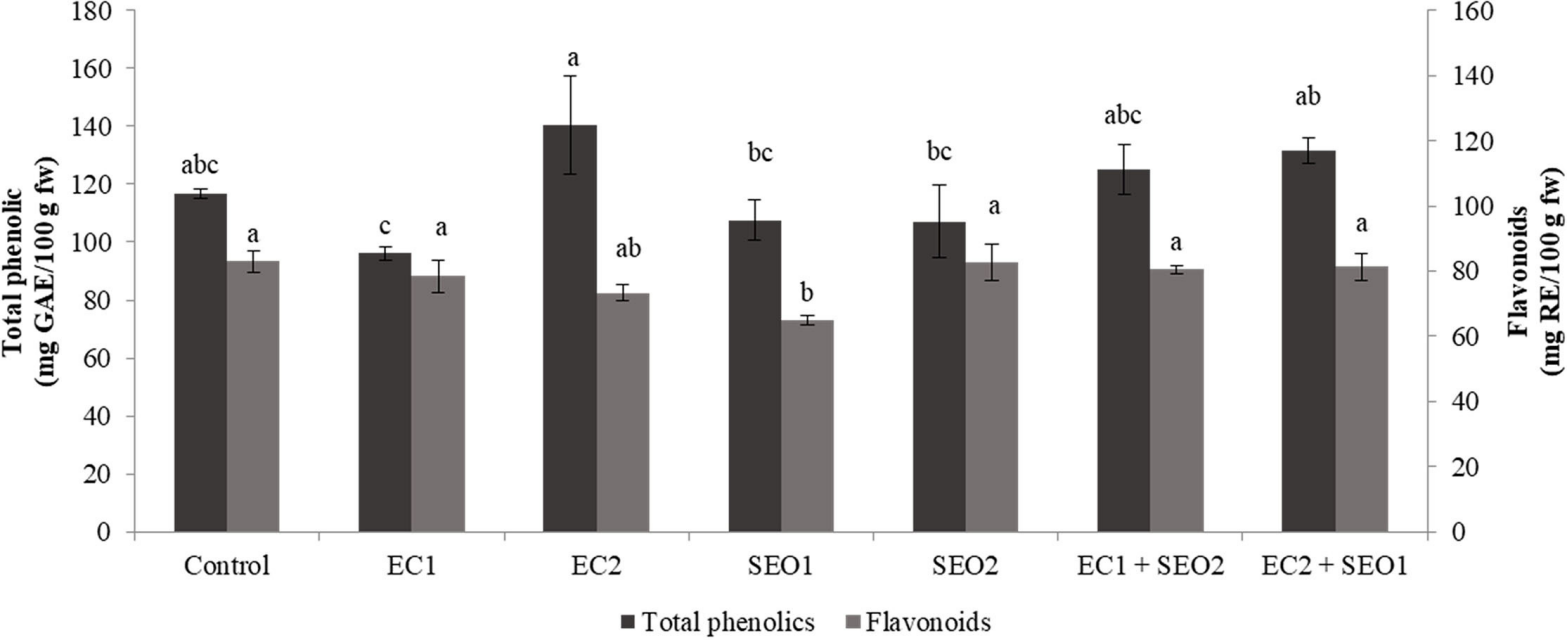
Total phenolics (mg GAE/kg fw) and flavonoids (mg RE/kg fw) content of tomato fruits under control (C, 0.4 dS/m), saline (EC1, 3.5 dS/m; EC2, 7 dS/m), spent engine oil stress (SEO1, 0.5%; SEO2, 1%) and the combinations (EC1/SEO2, 3.5 dS/m EC/1% SEO), (EC2/SEO1, 7 dS/m EC/0.5% SEO). Values are means (*n* = 3).

Phenolics are secondary metabolites widely distributed in plants and characterized by the presence of mono- or poly-hydroxylated aromatic rings. Plants contain tens of thousands of different phenolic compounds, and the number is still increasing, ranging from hydrophilic, lipophilic, to insoluble structures. Phenolics are categorized into groups and sub-groups based on the number of C-atoms and the fundamental arrangement of carbon skeletons in their chemical structure. They may act as antioxidants, structural polymers, coloring pigments, pollinator or pest chemo-attractants/repellents, UV screens, signaling molecules in symbiosis initiation, and plant-microbe interactions, and defensive weapons against aggressors (49). In this study, total phenolic and flavonoid contents varied significantly between the applied treatments (*P* <0.05) within ranges of 96.14–140.23 mg GAE/kg fw and 73.05–93.33 mg RE/kg fw (Figure 4). Compared to the control, SEO exposure, individually and in combination with EC stress, resulted, respectively, in a 7–8% decrease and a 6–21% increase in the content of total phenolics. However, contradictory effects of EC treatments were observed for the total phenolic content as fruits subjected to EC1 and EC2 treatments exhibited a 17% decrease and 20% increase compared to the control, respectively. ECs and SEO, individually and combined, negatively affected the flavonoid concentration, resulting in a 0.3–21% reduction. Phenolic compounds are important free radical scavengers, whose biosynthesis is significantly stimulated by various environmental stresses (50). Contrasting reports are available on the effect of salinity on phenolics. According to Akladious and Mohamed (51), a moderate increase (from 91.5 to 98.0 mg GAE/100 g fw) in the levels of total phenolics was observed following irrigation of peppers with a 100 mmol/L saline solution. Botella et al. (8) noticed a chemical-class dependent-response of tomato phenolics under salinity stress, with flavanones not affected but flavonols significantly increased, suggesting a specific role of the latter in scavenging salt-stress generated ROS. Moles et al. (41) noticed higher tolerance/resistance to increasing concentration (0–120 mm NaCl) of salt in tomato landraces (Ciettaicale, Linosa, and Corleone) grown in hydroponics, with respect to the commercial cv. UC-82B, which correlated with a differential accumulation of glycoalkaloids, phenolic acids, flavonoids, and their derivatives in the fruits. Yildiztugay et al. (52) proposed that exogenously applied flavonoid naringenin protects bean chloroplasts minimizing salinity-induced stress and increasing the activities of various antioxidant enzymes (ascorbate peroxidase, glutathione reductase, monodehydroascorbate reductase, dehydroascorbate reductase, and peroxidase).

In the case of SEO stress, the data on fruit quality is scarce, nevertheless, Muzolf-Panek et al. (48) reported a strong genotype-dependent increase in the content of total phenolic compounds (up to 11-fold) in two tomato cvs (Emotion F1 and Alboney F1) exposed to Mn (0.3 and 19.2 mg/dm^3^). Aguebor-Ogie et al. (53) noticed an organ-dependent response to spent lubrification oil stress in 2-week-old tomato seedlings. In the stem, total phenolic content was decreased by 0.6% but flavonoids increased by 84%, while in the root, total phenolic content was unaffected and total flavonoids decreased by 35% compared to the control. Molina and Seguna (32) proposed that the stimulation of phenylalanine ammonia-lyase activity and the concentration of the precursor's phenylalanine, tyrosine, and tryptophan increased following heavy-metal treatments highlighting the importance of phenolic compounds in counteracting stress-generated ROS.

### Total Vitamin C

**Figure 5:**
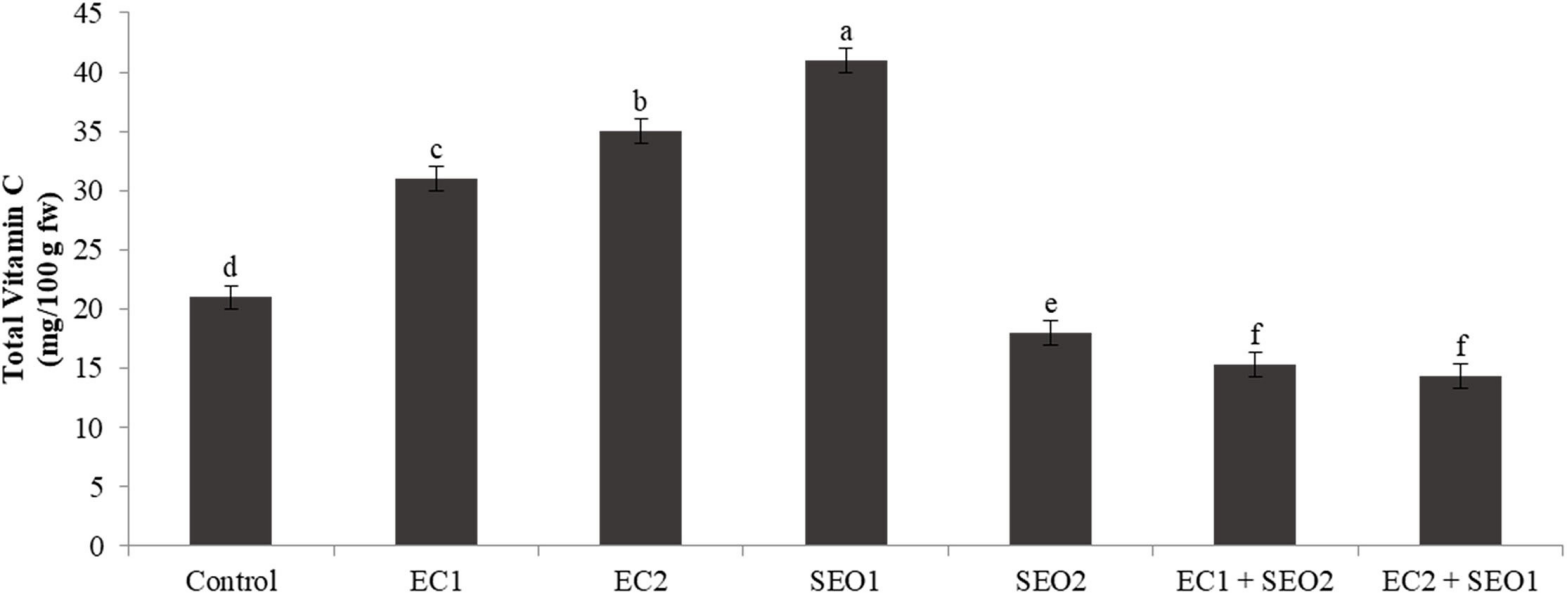
Total vitamin C content (mg/kg fw) of tomato fruits under control (C, 0.4 dS/m), saline (EC1, 3.5 dS/m; EC2, 7 dS/m), spent engine oil stress (SEO1, 0.5%; SEO2, 1%) and the combinations (EC1/SEO2, 3.5 dS/m EC/1% SEO), (EC2/SEO1, 7 dS/m EC/0.5% SEO). Values are means (*n* = 3).

Vitamin C has a pivotal role in maintaining redox homeostasis in plants. It acts directly as antioxidants scavenging reactive oxygen species, but can also regenerate glutathione and tocopherol radicals or acts as a cofactor for many enzymes (e.g., ascorbate peroxidases and violaxanthin de-epoxidase in the xanthophyll cycle) (54). In this study, the levels of total vitamin C were significantly (*P* <0.05) but differentially affected by the applied treatments (Figure 5). Compared with the control, EC treatments induced a concentration-dependent increase in total vitamin C levels (47–66%), which was, instead, decreased (up to −31%) when in combination with SEO. Individual SEO applications determined contrasting responses: 95% higher levels of total vitamin C were registered under SEO1, while a 14% decrease resulted from SEO2 treatment. The content of ascorbic acid was reduced in the stems and roots of 3-week-old tomato seedlings exposed to spent lubrification oil, from 2.28 to 1.52 mg/g and from 1.87 to 1.25 mg/g, respectively (53). Muzolf-Panek et al. (48) reported a significant inverse correlation between Mn concentration and total vitamin C levels in ripe fruits of the tomato cvs Emotion F1 (R = −0.603) and Alboney F1 (R = −0.668).

### Tocopherol Content

**Figure 6:**
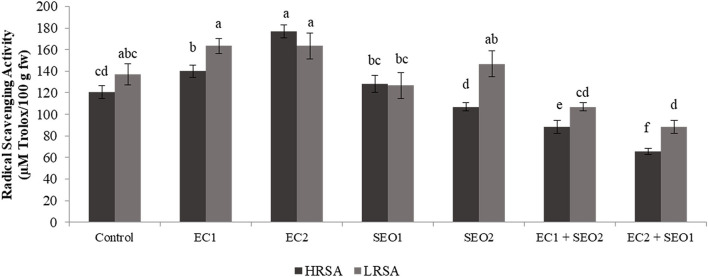
α-, β-, and γ-ocopherol content (mg/kg fw) of tomato fruits under control (C, 0.4 dS/m), saline (EC1, 3.5 dS/m; EC2, 7 dS/m), spent engine oil stress (SEO1, 0.5%; SEO2, 1%) and the combinations (EC1/SEO2, 3.5 dS/m EC/1% SEO), (EC2/SEO1, 7 dS/m EC/0.5% SEO). Values are means (*n* = 3).

Tocopherols play a crucial role in the protection of plants against oxidative stress, with mechanisms that may vary depending upon its severity and duration. Under moderate stress or in the early phase of severe stress, tocopherols act as typical antioxidants, while in the late phase of severe stress they may assist the recovery and recycling of vital compounds for the plant (55). In addition, it is widely recognized that the increase in tocopherol content contributes to plant stress tolerance while a decrease is associated with stress susceptibility (56). In this study, α-, β-, and γ-tocopherol contents varied significantly among the applied treatments (*P* <0.05) (Figure 6) within the ranges 10.23–31.69 mg/kg fw, 0.00–0.56 mg/kg fw, and 0.00–0.39 mg/kg fw, respectively. Compared to the control, EC and SEO individual treatments resulted in a 26–31% increase and a 19–56% decrease in the content of α-tocopherol, respectively. β-Tocopherol biosynthesis seems completely inhibited by SEO treatments when applied individually or in combination with ECs, and also by the EC2 treatment; however, a 93% increase in β-tocopherol concentration was observed under EC1. Similarly, a 24–86% decrease of γ-tocopherol content was observed in the fruits harvested from SEO-treated tomato plants, while it was not detected under both EC/SEO combined treatments. EC1 induced an 18% increase in γ-tocoperol but EC2 had a 26% reduction compared to the control. Spicher et al. (57) performed a lipidomic study to assess the effect of combined high temperature and light stress on *vte5* transgenic Microtom tomato plants and found that α-tocopherol and plastoquinone/plastoquinol biosynthetic pathways were strongly upregulated, among hundreds of targeted compounds, in response to the applied stresses. The authors concluded that VTE5 protects against combined high-light and high-temperature stress by positively modulating α-tocopherol production.

### Hydrophilic and Lipophilic Radical Scavenging Activity

**Figure 7:**
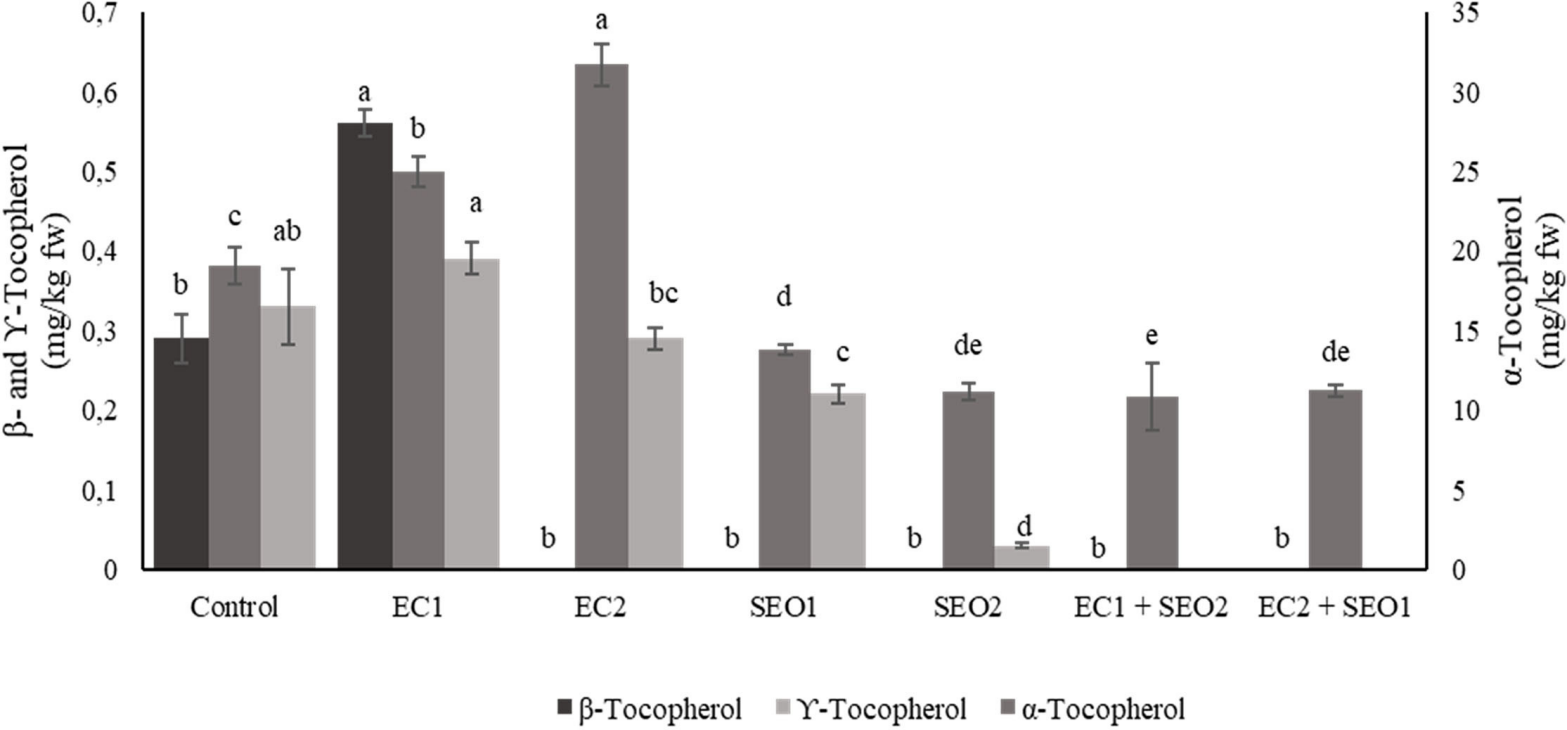
HRSA and LRSA (μM Trlox/100 g fw) of tomato fruits under control (C, 0.4 dS/m), saline (EC1, 3.5 dS/m; EC2, 7 dS/m), spent engine oil stress (SEO1, 0.5%; SEO2, 1%) and the combinations (EC1/SEO2, 3.5 dS/m EC/1% SEO), (EC2/SEO1, 7 dS/m EC/0.5% SEO). Values are means (*n* = 3).

HRSA and LRSA varied significantly between the applied treatments (*P* <0.05) within the values 65.3–176.7 and 68.3–163.4 μm Trolox/100 g fw (Figure 7) EC and SEO treatments applied individually resulted in 16–26% increase and 16–28% decrease in the HRSA values, respectively, compared with the control, EC treatments applied individually or in combination with SEO resulted in 19.0–19.3% increase and 21–35% decrease in LRSA values, respectively. However, SEO applied individually induced conflicting responses: LRSA was reduced by 7.0% by SEO1 and increased by 7.1% by SEO2. The values obtained in control conditions are similar (116.7 μm trolox/100 g fw to 279.4 μm trolox/100 g fw) to our previous reports for different open-field tomato cvs (5, 39). Sumalan et al. (9), assessing the antioxidant profile of some salt-tolerant tomato landraces, found that the best cultivars in terms of functional quality were those grown under high concentrations of soil salinity ranging from 7.21 dS/m-6.58 dS/m such as landraces CN-254 and L-189b, respectively. Tommonaro et al. (58) assessed the phytochemical and nutritive features of the pulp and seeds from tomato fruits grown in muddy soils revealing high values of antioxidant activity, especially in the lipophilic fraction, with the absence of heavy metals and cytotoxic effect in both fractions. Muzolf-Panek et al. (48) reported that the antioxidant activity significantly increased with increasing Mn concentration (1.2–2.4 mg/dm^3^) in tomato cvs Emoticon F1 and Alboney F1 fruits grown in hydroponics. Dursun (59) assessed the activities of ascorbate peroxidase, peroxidase, and superoxide dismutase in the leaves and roots of tomatoes cultivated under heavy metal (Cd, Cu, and Pb at 10, 20, and 50 ppm)-induced stress. The authors reported that ascorbate peroxidase activity in tomato roots changed depending on the heavy metal types and concentrations.

The response to SEO treatments may vary depending not only on treated plant species but also on other aspects including pollutant type, local concentrations, water solubility, plant age at the time of SEO contamination, and soil organic matter content and texture (60, 61).

The effect of combined stresses can be different from those of stresses applied individually, which suggests the importance to focus on how the interaction between stresses can affect the processing attributes and functional quality of tomato fruits.

### Correlation Study

Many authors have examined the correlations between and among phytochemicals secondary metabolites and antioxidant activities in several fruits and vegetables, including tomatoes (5, 39). However, little is known concerning tomato cvs subjected to stress. The Pearson's correlation coefficients of all the assayed processing and functional attributes of tomato fruits subjected to EC and SEO individual and combined treatments are reported in Table 3. A significant positive correlation was evidenced between HRSA and β-carotene, lycopene, total vitamin C, α-tocopherol, γ-tocopherol, average fruit weight, soluble solids, and a^*^/b^*^ ratio, as well as between LRSA and β-carotene, lycopene, α-tocopherol, γ-tocopherol, average fruit weight, soluble solids, and a^*^/b^*^ ratio. Titratable acidity negatively correlated with both HRSA and LRSA. Interestingly, a^*^/b^*^ ratio was positively and significantly (*P* <0.01) correlated with β-carotene (R = 0.78), lycopene (R = 0.68), total vitamin C (R = 0.71), α-tocopherol (R = 0.83), γ-tocopherol (R = 0.66), HRSA (R = 0.93), LRSA (R = 0.80), and soluble solids (R = 0.84), and can thus represent a good indicator of fruit quality even in fruits subjected to soil salt and SEO pollution.

**Table 3:** Pearson correlation coefficients of β-carotene (β-Crt), lycopene (Lyc), total phenolic compounds (TPC), total flavonoids (TF), total vitamin C (TVC), α-, β-, and γ-Tocopherol (α-, β-, and γ-T), hydrophilic radical scavenging activity (HRSA) and lipophilic radical scavenging activity (LRSA), total soluble solids (TSS), titratable acidity (TA), a^*^/b^*^ ratio, average fruit weight (Avg. fruit wt), and yield per plant (Y/pl).

**Traits^**a**^**	**β-Crt**	**Lyc**	**TPC**	**TF**	**TVC**	**α-T**	**β-T**	**⋎-T**	**HRSA**	**LRSA**	**TSS**	**TA**	(a*/b*)	**Avg. fruit wt**.	**Y/pl**.
β-Crt	1														
Lyc	0.76**	1													
TPC	−0.08^ns^	0.014^ns^	1												
TF	−0.49*	−0.49*	0.13^ns^	1											
TVC	0.76**	0.79**	−0.16^ns^	−0.68**	1										
α-T	0.49*	0.58**	0.11^ns^	−0.19^ns^	0.57**	1									
β-T	−0.27^ns^	−0.37^ns^	−0.084^ns^	0.16^ns^	−0.12^ns^	0.16^ns^	1								
γ-T	0.41^ns^	0.39^ns^	−0.26^ns^	−0.28^ns^	0.66**	0.78**	0.50*	1							
HRSA	0.77**	0.70**	−0.04^ns^	−0.38^ns^	0.76**	0.86**	0.11^ns^	0.75**	1						
LRSA	0.63**	0.51*	−0.22^ns^	0.05^ns^	0.48*	0.64**	0.13^ns^	0.63**	0.79**	1					
TSS	0.65**	0.73**	−0.02^ns^	−0.52*	0.87**	0.83**	−0.02^ns^	0.76**	0.87**	0.61**	1				
TA	−0.41^ns^	−0.1^ns^	0.36^ns^	0.12^ns^	−0.45*	−0.50*	−0.56**	−0.82**	−0.60**	−0.63**	−0.47*	1			
a*/b*	0.78**	0.68**	−0.07^ns^	−0.33^ns^	0.71**	0.83**	−0.05^ns^	0.66**	0.93**	0.80**	0.84**	−0.55*	1		
Avg. fruit wt.	0.30^ns^	0.09^ns^	−0.25^ns^	−0.03^ns^	0.39^ns^	0.66**	0.66**	0.88**	0.64**	0.62**	0.54*	−0.92**	0.56**	1	
Y/pl.	0.19 ^ns^	−0.71 ^ns^	−0.19 ^ns^	0.70 ^ns^	0.29	0.51*	0.66**	0.78**	0.51*	0.51*	0.36 ^ns^	−0.88**	0.40 ^ns^	0.91**	1

ns = non-significant; ^*^, ^**^ = significant at P <0.05 or 0.01, respectively.

## Conclusion

In summary, EC treatments applied individually reduced average fruit weight but increased soluble solids, titratable acidity, a^*^/b^*^ ratio, β-carotene, lycopene, total vitamin C, α-tocopherol, HRSA, and LRSA. SEO stress decreased average fruit weight, HRSA, α-tocopherol, γ-tocopherol, total phenolics, and flavonoids, but increased titratable acidity, a^*^/b^*^ ratio, β-carotene, and lycopene. Similarly, to the processing attributes, our findings point out that the response to EC1/SEO2 and EC2/SEO1 combined treatments was idiosyncratic for β-carotene and dominated by SEO for lycopene, respectively; dominated by SEO and EC, respectively, for total phenolic, dominated by EC for flavonoids, and dominated by SEO and idiosyncratic for total vitamin C. An idiosyncrasy was observed for α- and γ-tocopherols under EC1/SEO2 and EC2/SEO1, respectively. However, the response was dominated by SEO for β-tocopherol. Regarding the RSA, the response was dominated by SEO and idiosyncratic for HRSA and totally idiosyncratic for LRSA. Although the alteration affecting the processing and functional quality of tomato fruits grown under EC and/or SEO, the produced fruits exhibited increased levels in various metabolites under moderate salinity stress, including β-carotene, lycopene, total phenolics, total vitamin C, tocopherols as well as the HRSA and LRSA. Under (EC2/SEO1, HRSA, and LRSA were severely affected and decreased by more than 25%. In addition, the a^*^/b^*^ ratio was positively and significantly correlated with most assayed functional attributes metabolites and total soluble solids suggesting that this stress-induced fingerprint may be used to detect early soil contamination to avoid hazard compounds contamination in the food chain.

## Data Availability Statement

The original contributions presented in the study are included in the article/supplementary material, further inquiries can be directed to the corresponding author.

## Author Contributions

RI, IT, and TR'h conceptualized the study and were in charge of tomatoes cultivation, fruit sampling, and reagent preparation. HD, RI, ZP, and LH contributed to the study conception and design, optimization of HPLC analysis, and made valuable recommendations and suggestions for HPLC peaks integration and the calculation of different bioactive compounds contents. ML, MS, MA, ZP, MD, and AM did statistical analysis, interpretation of data made recommendations, and suggestions regarding the article. All the authors contributed directly or indirectly to the study conception and design and interacted positively during the preparation of this article.

## Funding

This research was supported by the Ministry of Innovation and Technology within the framework of the Thematic Excellence Programme 2020, Institutional Excellence Sub-programme (TKP2020-IKA-12), and the EFOP-3.6.3-VEKOP-16-2017-00008 project.

## Conflict of Interest

The authors declare that the research was conducted in the absence of any commercial or financial relationships that could be construed as a potential conflict of interest.

## Publisher's Note

All claims expressed in this article are solely those of the authors and do not necessarily represent those of their affiliated organizations, or those of the publisher, the editors and the reviewers. Any product that may be evaluated in this article, or claim that may be made by its manufacturer, is not guaranteed or endorsed by the publisher.
